# U12, a UDCA Derivative, Acts as an Anti-Hepatoma Drug Lead and Inhibits the mTOR/S6K1 and Cyclin/CDK Complex Pathways

**DOI:** 10.1371/journal.pone.0113479

**Published:** 2014-12-08

**Authors:** Yang Xu, Qiang Luo, Ting Lin, Zhiping Zeng, Guanghui Wang, Dequan Zeng, Rong Ding, Cuiling Sun, Xiao-kun Zhang, Haifeng Chen

**Affiliations:** 1 School of Pharmaceutical Sciences, Xiamen University, Xiamen, Fujian, PR China; 2 Sanford-Burnham Medical Research Institute, La Jolla, California, United States of America; Florida State University, United States of America

## Abstract

U12, one of 20 derivatives synthesized from ursodeoxycholic acid (UDCA), has been found to have anticancer effects in liver cancer cell lines (SMMC-7721 and HepG2) and to protect normal liver cells from deoxycholic acid (DCA) damage (QSG-7701). Its anticancer mechanism was investigated using computer-aided network pharmacology and comparative proteomics. Results showed that its anti-malignancy activities were activated by mTOR/S6K1, cyclinD1/CDK2/4 and caspase-dependent apoptotic signaling pathways in hepatocellular carcinoma cells (HCC). The action of U12 may be similar to that of rapamycin. Animal testing confirmed that U12 exerted better anti-tumor activity than UDCA and had less severe side effects than fluorouracil (5-Fu). These observations indicate that U12 differs from UDCA and other derivatives and may be a suitable lead for the development of compounds useful in the treatment of HCC.

## Introduction

Hepatocellular carcinoma (HCC) accounts for 75–90% of all cases of liver cancer in most countries. HCC is the sixth most common cancer in the world and it is especially common in Africa, southeast Asia, and China [1]. The majority of cases of HCC arise against a background of chronic liver disease, including hepatitis B virus (HBV) and hepatitis C virus (HCV), or ethanol abuse. Recently, epidemiologic investigations have indicated that the incidence and mortality rate of HCC is growing in the U.S. and some European countries [1], [2]. Although these factors have intensified research efforts into new treatment strategies, there still are few effective drugs without drug resistance. Currently, sorafenib (previously known as BAY 43-9006) is the only drug approved for the treatment for HCC by the Food and Drug Administration of the United States.

Ursodeoxycholic acid (UDCA) is a secondary bile acid produced by intestinal bacteria. It has been used as a therapeutic agent in cholestatic liver disease, primary biliary cirrhosis (PBC), and primary sclerosing cholangitis (PSC) [3], [4]. Although extensive investigations have been performed on UDCA, the biochemical mechanism underlying its effects is still not well understood. In clinical settings, administration of UDCA to PBC patients causes significant improvement in liver biochemistry. UDCA therapy also has been shown to delay the progression of liver fibrosis and to reduce the development of severe liver disease while fostering improvement of serum liver enzymes [5]. In addition, UDCA exhibits anti-apoptotic effects in both hepatocytes and non-hepatic cells and has a pronounced effect on the prevention of colon cancer [6]–[8]. It exerts this effect through several mechanisms [9], [10]. For these reasons, UDCA derivatives have captured a significant amount of attention. UDCA-glutamate (UDCA-Glu) shows little intestinal absorption, resulting in increased colonic delivery, which enhances the effects of UDCA [11]. NCX 1000, a nitric-oxide-releasing derivative of UDCA (UDCA-NO), has been found to protect hepatocytes from acetaminophen-induced toxicity and to prevent the development of portal hypertension through the selective release of NO in the liver, the maintenance of mitochondrial integrity, and further inhibition of apoptosis [12], [13]. The UDCA derivative HS-1183 has also been shown to exert anti-tumor effects. This induced apoptosis and inhibited the proliferation of human breast and prostate cancer cell lines through a p53-independent/p21-dependent pathway and prevents the death of HS-1183-induced human cervical carcinoma cells via nuclear translocation of nuclear factor (NF)-kappa B and activation of c-Jun N-terminal kinase [14]–[16]. Considering the original use of UDCA in liver disease and the small number of intensive studies that have been performed on the anti-hepatoma effect of UDCA derivatives, it is here hypothesized that UDCA derivatives may be a suitable anti-hepatoma chemotherapeutic reservoir.

Because of the anti-apoptotic effects of UDCA, a series of UDCA derivatives,including U12, were synthesized for the further screening. Bioinformatics and proteomic strategies were combined and used to identify the pathways possibly involved in U12-associated anticancer effects. Biochemical approaches and animal testing were used to determine how U12 affected cancer cell apoptosis and prevented proliferation in HCC.

## Materials and Methods

### Ethics statement

The study was approved by the Laboratory Animal Management and Ethics Committee of Xiamen University, China. Mice were housed according to sex and genotype, 4 per cage and maintained on a 12 hour light: dark cycle (lights on at 7:00am) with continuous access to food and water.

### Cell culture and drug treatment

HepG2, SMMC-7721, and QSG-7701 cells were obtained from the Chinese Academy of Sciences Cell Bank [17]. They were cultured in Dulbecco's Modified Eagle Medium (high glucose) plus 10% fetal bovine serum (JRH Bioscience, Lenexa, KS, U.S.) under standard culture conditions. When the cells reached about 80% confluence, they were subcultured or treated with drugs as necessary. After treatment, the cells were washed twice with PBS. Protein concentration was determined using BCA. In the caspase inhibitor assay, cells were treated with 50 µM Z-VAD-fmk or 20 µM Z-IETD-fmk for 1 h before U12 treatment. Antibodies, inhibitors, and other chemicals were purchased from Sigma-Aldrich Chemicals, except otherwise noted.

### Growth inhibition analysis

The proliferation of cells was assessed using MTT [3-(4,5-dimethylthiazol-2-yl)-2, 5-diphenyl tetrazolium bromide]. The MTT assay was based on the conversion of MTT to MTT-formazan by mitochondrial enzyme, as described [18] and calculated [19] previously. 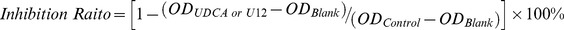



### Caspase activity

SMMC-7721 cells were collected and processed with Caspase-3/CPP32, FLICE/Caspase-8, and Caspase-9 Colorimetric Assay Kits as described in the manufacturer's protocol (Biovision Research Products). The chromophore p-nitroanilide, cleaved from the corresponding substrates (DEVD-pNA, IETD-pNA, and LEHD-pNA) was detected using a spectrophotometer at 405 nm as needed. Data were normalized to protein content and are shown relative to the activity observed in the untreated control.

### Flow cytometry for cell cycle distribution

SMMC-7721 cells were treated with the desired doses of U12 for 12 or 24 h. At the end of each experiment, 1×10^6^ SMMC-7721 cells were harvested, stained with PI, and then analyzed using a FAC Plus flow cytometer. Cell cycle distribution was determined in each treatment group using WinMDI 2.9.

### MetaDrug analysis

The molecular structure of U12 was uploaded into MetaDrug All the original settings were used. Target identification and pathway prediction were performed through a similarity search by GeneGo Pathway Maps and GeneGo Disease Biomarker Networks.

### 2DE and MS analysis

2DE was performed using GE IPGphor III and Ettan Dalt Six (GE Healthcare) electrophoresis units, a following protocol that has been described previously [18]. Image analysis was carried out with ImageMaster 2D platinum software (GE Healthcare). Only protein spots that showed reproducible alterations in three independent experiments (over 2-fold up- or down-regulation, *P*<0.05) were selected for tryptic in-gel digestion and MS analysis. Peptide mass spectra were obtained on an ABI 5800 MALDI TOF/TOF mass spectrometer in a reflector positive ion mode with an average of 1500 laser shots per spectrum. Peptide ion masses were internally calibrated using a mass standards kit at m/z 904.46, 1296.68, 1570.67, 2093.08, and 2465.19. TOF/TOF tandem MS fragmentation spectra were acquired in a data-dependent fashion based on the MALDI-TOF peptide mass map for each protein. The 10 most abundant ions (excluding trypsin autolytic peptides and other known background ions) present in each sample were selected. All these data were processed using GPS Explorer software (V3.6, Applied Biosystems). MASCOT was used to identify the proteins using NCBInr protein database. Species searches were limited to humans.

### Western immunoblotting

Total cell lysates were denatured with sample loading buffer and then subjected to SDS-PAGE for protein separation. After transfer to membranes, the proteins were probed with corresponding antibodies and detected with ECL detection reagents (GE). The primary antibodies against mTOR (2983P), p-mTOR (2971S), S6K1 (2708P), p-S6K1 Ser371 (9208P) and Thr389 (9234P), PARP (9542), Rb (9313S), p-Rb Ser807 (8516P) and Ser795 (9301P), cyclin D1 (2926P), CDK4 (2906P), CDK6 (3136P) and p27 (2552P) were obtained from Cell Signaling (Beverly, MA, U.S.). Primary antibodies for Cdc25A (sc-4256) and LaminA/C (sc-7293) were purchased from Santa Cruz Biotechnology (Santa Cruz, CA, U.S.). Anti-β-actin (A4700) was obtained from Sigma. Secondary anti-mouse and rabbit antibodies were purchased from Thermo.

### Animal testing

Each experimental group contained eight male nude mice. HepG2 cells were subcutaneously implanted into these mice when they were 6 weeks old. When the tumors became palpable, which happened just under 2 weeks after implantation, the mice were treated intraperitoneally with vehicle control (2% DMSO in maize oil), 30 mg/kg 5-Fu or 250 mg/kg U12 every day for 2 weeks. Tumor volume and mouse weight were assessed every two days. Tumor volumes were calculated using the following formula: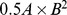, where “A” is the long diameter and “B” is the short diameter of the tumor measured using calipers (cm) [20]. At the end of the treatment (14 days), mice were euthanized and weighed.

### Statistical analysis

Statistical significance was calculated using a two tailed Student's t-test and *P*<0.05 was considered significant. Data are expressed as mean ±SD and all cases are representative of at least 3 independent studies.

## Results

### Chemical synthesis of UDCA derivatives

**Figure 1:**
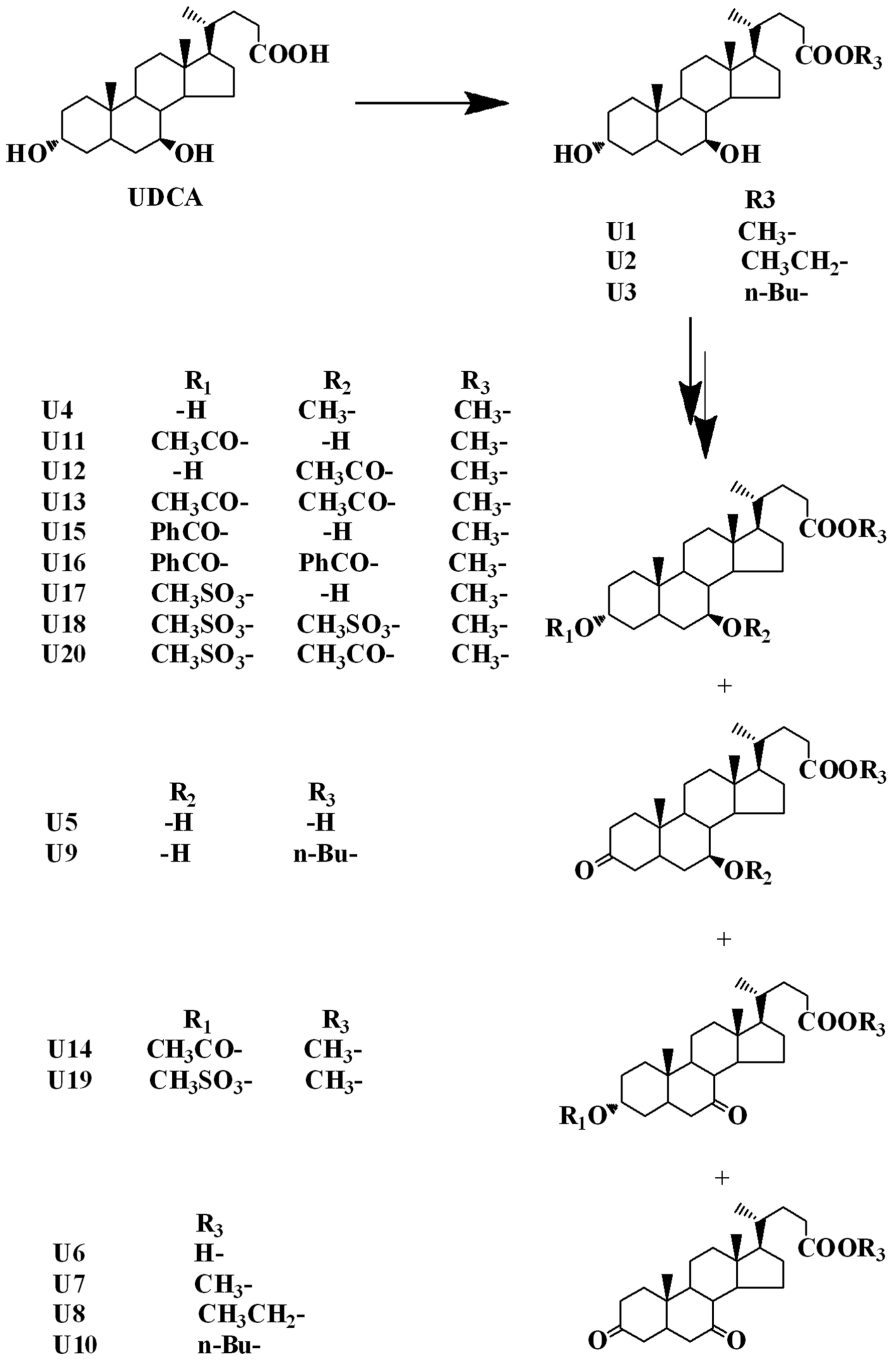
Chemical structures of UDCA and its derivatives.

Twenty different UDCA derivatives were obtained via synthesis. NMR spectra were used for structural identification. UDCA was esterified using the corresponding alkanol (methanol, ethanol or n-butanol) and oxidized to produce U1–3 and U5–6; U1 was etherified, oxidized, esterified, and sulfonated to form U4, U7, U11–13 and U15–18; U2 was oxidized to produce U8 and U3 was oxidized to produce U9–10; U11 was esterified to produce U14; U17 was oxidized and esterified to produce U19–20. The details of the structures and synthetic routes are shown in Fig. 1 and S1 File.

### Cytotoxicity of UDCA derivatives to normal and liver cancer cell lines

**Figure 2:**
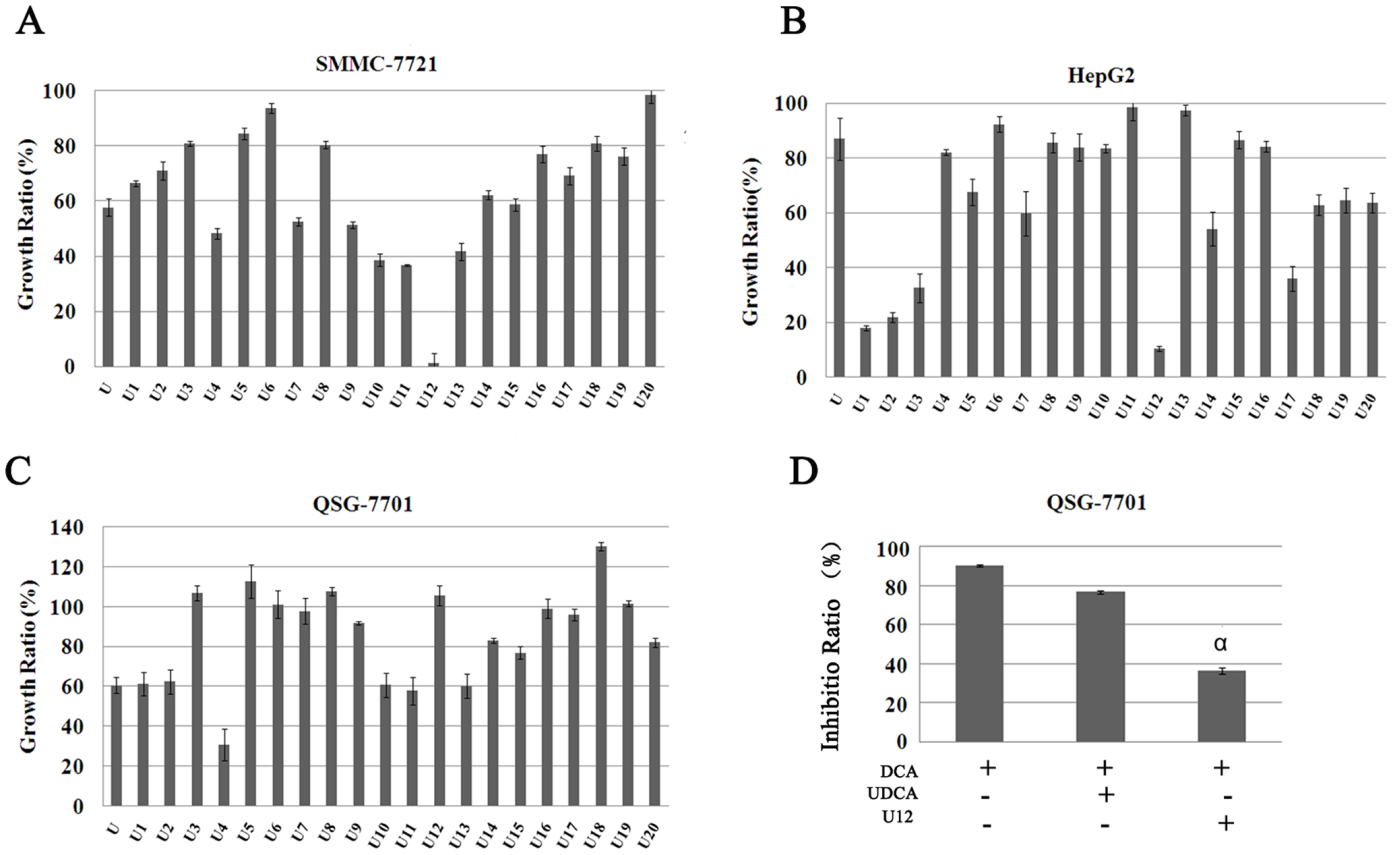
Evaluation of UDCA and its derivatives effects on different cell lines. The growth ratio of UDCA and its 20 different derivatives on (**A**) SMMC-7721, (**B**) HepG2, and (**C**) QSG-7701 were detected by MTT assay. (A–C shows the ratios relative to untreated controls). All compounds were administered at concentrations under 100 µM and allowed to incubate for 24 h. (**D**) QSG-7701 cells were either untreated or pretreated with 100 µM UDCA and U12 for 18 h. The cultures were replaced with 300 µM DCA and allowed to incubate for 6 h and then an MTT assay was performed to assess the ability of UDCA and U12 to rescue cytotoxicity induced by DCA. Results are representative of three independent experiments, showing mean±SD (α, *P*<0.05, compared with UDCA treatment).

An MTT assay was used to investigate the effects of UDCA and its derivatives on the viability of SMMC-7721, HepG2, and QSG-7701 (Fig. 2A–C). Cell growth ratios in experimental groups and controls were assessed after administration of 100 µM UDCA and its derivatives for 24 h. U12 showed the most pronounced cytotoxicity toward both liver cancer cell lines (SMMC-7721 and HepG2). Considering UDCA can antagonize DCA-induced impairment to different extents depending on the conditions, whether U12 may prevent the action of DCA was here evaluated [21], [22]. Results showed that U12 can improve DCA-induced cell inhibition by more than 60% in QSG-7701 cell lines. U12 provided significantly more protection than UDCA (*P*<0.05) (Fig. 2D). All these results showed that U12 can induce both liver cancer cell death and protect normal liver cells from DCA treatment. For this reason, U12 was selected for further investigation.

### Extrinsic apoptotic characteristics of SMMC-7721 cells in the presence of U12

**Figure 3:**
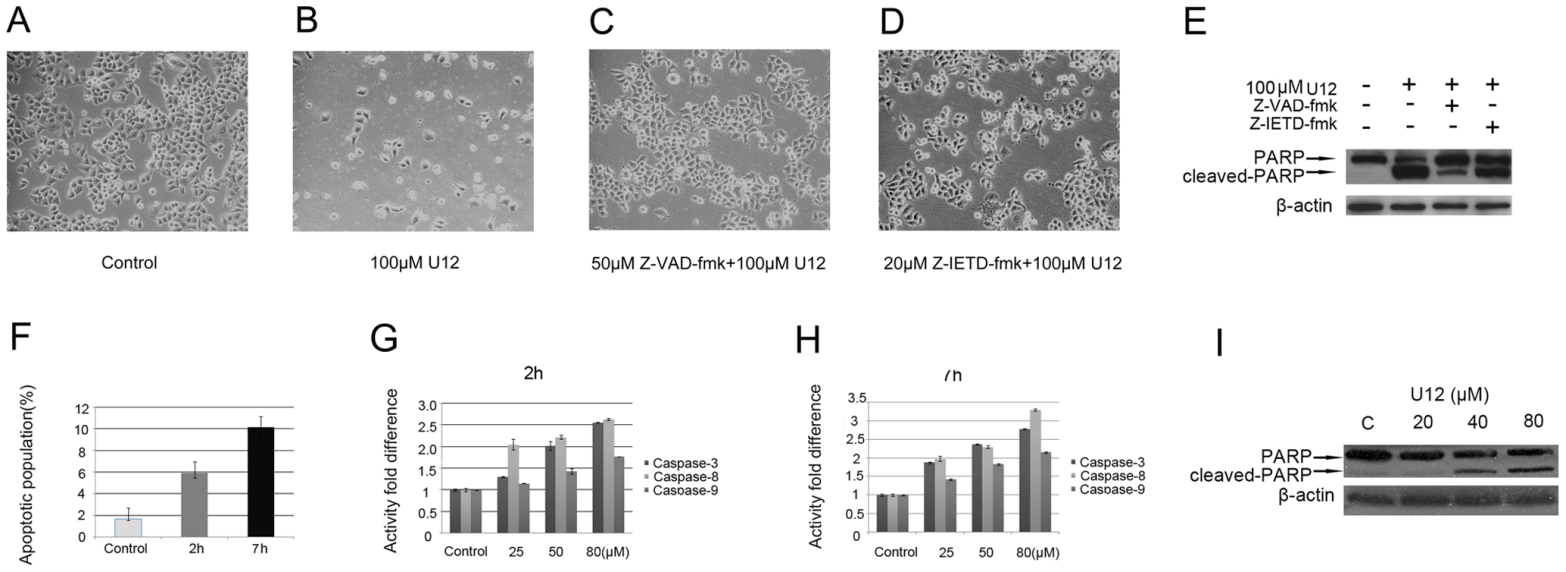
U12-induced apoptosis in SMMC-7721. Morphological and quantitative changes in SMMC-7721 cells after being (**A**) left untreated, (**B**) treated with 100 µM U12 for 24 h, or (**C**) pretreated with 50 µM Z-VAD-fmk or (**D**) 20 µM Z-IETD-fmk for 1 h. (**E**) Western blotting was used to estimate PARP cleavage from the 100 µM U12 for 24 h treatments. (**F**) Detection of apoptotic SMMC-7721 cells in the presence of 80 µM U12 for 2 h and 7 h using Annexin V-FITC/PI analysis. (**G**&**H**) Activation of caspase-3, -8, and -9 was evaluated using a caspase activity kit after indicated concentration of U12 treatment at 2 h and 7 h, respectively. (**I**) Western blot analysis of PARP cleavage on SMMC-7721 cells untreated and treated with indicated concentration of U12 at 12 h.

After treatment with U12 for 24 h, SMMC-7721 cells showed considerable changes in shape and number (Fig. 3A&B). Cells were further pretreated with 50 µM broad Z-VAD-fmk (spectrum caspase inhibitor) and 20 µM Z-IETD-fmk (specific inhibitors of caspase-8) for 1 h before U12 treatment. The samples pretreated with caspase inhibitors showed significantly more cell viability than those treated with U12 alone (Fig. 3A–D). These results were confirmed by Western blot analysis, indicating that caspase inhibitors could rescue the cells from U12-related apoptosis (Fig. 3E). Flow cytometric analysis was further used to determine whether U12 can induce apoptosis in SMMC-7721 cells. Double staining of Annexin V-FITC/propidium iodide (PI) was used to determine the number of apoptotic cells, which was used to assess the translocation of phosphatidylserine (PS) from the inner plasma membrane to the outer membrane (Annexin V FITC-positive, PI-negative). As shown in Fig. 3F, administration of U12 for 2 h resulted in a 4.26% increase in the number of apoptotic cells and the level continued to increase to 10.14% after 7 h of treatment. In addition, the time- and dose-course of U12-induced alterations in the caspase enzyme activities were measured using substrates specific to different caspases in *vitro*, including DEVD (caspase-3), IETD (caspase-8), and LEHD (caspase-9). The activation of caspase-3, -8, and -9 was tested. Early during treatment (2 h) at low U12 concentrations (25 µM), caspase-8 activity was found to be twice as pronounced as that of caspase-3 and -9 (Fig. 3G&H). Dose-related cleaved-PARP expression was also observed after U12 administration (Fig. 3I).

### Prediction of the mechanism of U12 anticancer action

MetaDrug is a leading systems pharmacology platform designed for the prediction and assessment of biological effects of small molecule compounds. Especially, it can be used to predict the properties based on the structure of individual newly synthesized compounds. To evaluate possible antineoplastic mechanisms, the chemical structure of U12 was loaded into MetaDrug software (GeneGo, Inc.). An enrichment analysis showed 7 of the top 20 predictive pathways to be associated with cell cycle regulation. Most of these pathways were involved in the G1 stage (Table 1 and S1 Figure). Further investigations should focus on U12-induced regulation of the G1 cell cycle. There are many pathways that could influence the G1 cell cycle. A comparative proteomic approach was applied to clarify and definite the proteins and pathways, which are involved in U12-associated G1 cell cycle arrest.

**Table 1:** Seven of the top 20 predictive pathways were found to be associated with U12-induced cell cycle regulation on SMMC-7721 cells.

NO.	Maps	-log(p-Value)
3	Cell cycle_Cell cycle (generic schema)	>1.75
4	Cell cycle_Role of 14-3-3 proteins in cell cycle regulation	>1.5
8	Cell cycle Role of SCF complex in cell cycle regulation	>1.5
13	DNA damage _ATM/ATR regulation of G1/S checkpoint	>1.5
14	Cell cycle_Role of APC in cell cycle regulation	>1.5
15	Cell cycle_ESR1 regulation of G1/S transition	>1.5
18	Cell cycle_Regulation of G1/S transition (part 1)	>1.25

### Alterations in cellular proteins in response to U12

**Figure 4:**
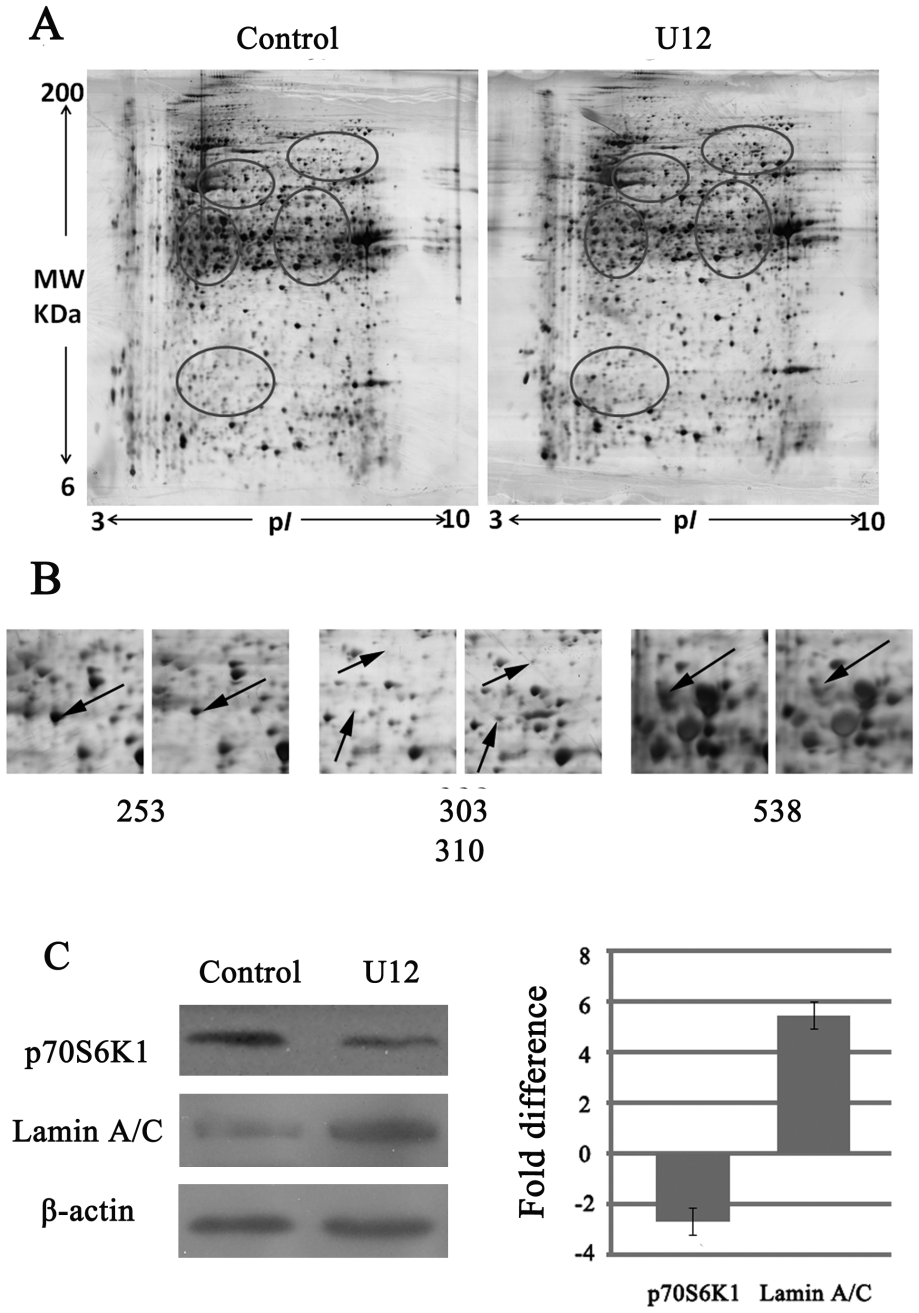
2DE analysis of U12-induced SMMC-7721 cell death. (**A**) 2-DE silver staining images of total proteins to untreated SMMC-7721 cells and cells treated with 100 µM U12 for 8 h. Representative images of 2-DE are from three independent experiments. (**B**) Altered protein spots related to U12-induced cell growth were identified using MS. (**C**) Western blots confirmation of the identified proteins from 2D-MS. Right: quantitative analyses, all data were normalized to the corresponding β-actin values and expressed as the percentage over the values obtained from the control groups. Bars represent average fold difference calculated from the three experiments.

Fig. 4A shows representative 2-dimensional electrophoresis (2DE) images for total proteins extracted from SMMC-7721 cells treated with U12 for 8 h and left untreated for the same length of time. More than 1000 protein spots were separated on the gel. These ranged in MW from 6–200 kDa and in p*I* from 3–10. The spots that showed considerable differences (>2-fold difference) from the untreated controls and U12 treatment samples were selected for matrix-assisted laser desorption-ionization time-of-flight mass spectrometry (MALDI-TOF MS) analysis to identify the proteins.

Within several categories of identified proteins (>20 altered proteins), the notable group was associated with the regulation of cell growth, including up-regulation of lamin A/C and elongation factor 2b (EF2B), partial-regulation and down-regulation of ribosomal protein S6 kinase (S6K1, also known as p70^S6K^), and far upstream element binding protein 1 (FBP1) (Fig. 4B). Table 2 lists proteins with spot ID numbers, name, GI number, MW/p*I* value, and fold differences between expression and scores. These alterations in protein expression suggested that U12 may exert a cytotoxic function through the pathways that interrupt normal regulation of the cell cycle. S6K1, the substrate of mammalian target of rapamycin (mTOR), was among the four most significantly altered proteins. mTOR is an important target of anti-tumor drug development [23], [24]. Biochemical methods can be used to determine the manner in which the cell cycle process is mediated by U12, especially mTOR/S6K1 related pathways. Fig. 4C displays the validation for the alterations of Lamin A/C and S6K1 using western blotting, which matched well with the 2DE and MS results.

**Table 2:** Protein alterations related to cell growth regulation in response to U12 treatment (100 µM for 8 h).

No. Spots	Protein Name	GI No.	Protein MW	Protein PI	Pep. Count	Protein Score	Protein Score C.I.%	Total Ion Score	Total Ion Score C.I.%	Fold Differences
253	ribosomal protein S6 kinase alpha-3	gi|4759050	84025.1	6.41	13	267	100	157	100	-2.63
303	elongation factor 2b, partial	gi|19353009	58147.7	6.51	15	311	100	233	100	+2.45
310	lamin A/C, isoform CRA_b	gi|119573384	65152.6	6.4	21	150	100	107	99.794	+5.39
538	far upstream element-binding protein 2	gi|154355000	73355.1	6.85	14	530	100	475	100	-3.12

### Cell cycle arrest of SMMC-7721 induced by U12

**Figure 5:**
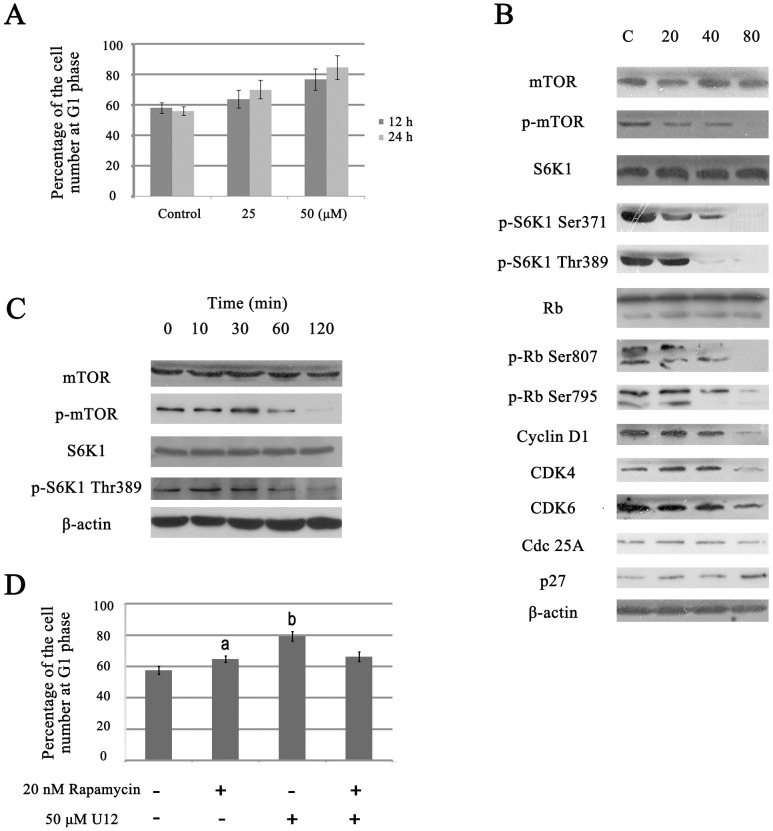
U12–associated cell cycle distribution in cancer cells. (**A**) G1 phase arrest induced by U12 in cell cycle progression of SMMC-7721 cells with various concentrations of U12 for 12 h and 24 h treatment was analyzed using flow cytometric analysis. (**B**) Western blot analysis of G1 cell cycle regulators (mTOR, p-mTOR Ser2448, p-S6K1 Ser371, p-S6K1 Thr389, PARP, p-Rb Ser807, p-Rb Ser 795, cyclin D1, CDK4, CDK6, and p27) under 24 h of U12 exposure at the indicated concentrations. (**C**) Western blot analysis of phosphorylated proteins (p- mTOR and p-S6K1 Thr389) within 2 h of 50 µM U12 administration. (**D**) G1 cell cycle arrest induced by treatment of U12 for 12 h with or without pretreated with rapamycin for 1 h on SMMC-7721 cells. (a, *P* = 0.479, relative to combination treatment with U12 and rapamycin; b, *P* = 0.007, relative to combination treatment with U12 and rapamycin). All of the results are representatives from three independent experiments.

The predictive data produced by MetaDrug analysis and proteomic research indicated that there have been interruptions in the growth of SMMC-7721 cells, especially G1 cell cycle arrest involving U12-induced cytotoxicity. Cell cycle progression after U12 treatment was evaluated through flow cytometry analysis. As shown in Fig. 5A, treatment with the indicated concentrations of U12 for 12 h and 24 h produced significant increases in the relative number of cells in the G1 phase. Administration of 25 µM and 50 µM U12 for 12 h or 24 h resulted in almost 6–28% elevation in the number of cells in the G1 phase (Fig. 5A). To determine the mechanism by which U12 induces G1 cell cycle arrest, the levels of expression of the proteins involved in the regulation of the G1 cell cycle were estimated. These proteins included cyclin and cyclin-dependent kinases (Fig. 5B). The mTOR/S6K1 pathway was also evaluated on the basis of proteomic research. Western blot analysis showed a strong decrease in the magnitude of reduction in phosphorylation in p-mTOR at Ser2448, p-S6K1 at Ser371 and Thr389 residues, p-Rb at Ser 807 and 795 residues; cyclin D1, cyclin-dependent kinase 4 (CDK4), CDK6, and Cdc25A, but there was no considerable change in total protein levels of β-actin or mTOR after 24 h of U12 treatment (Fig. 5B). The general trends of the phosphorylated mTOR and S6K1 Thr389 were reduced during short term observation at 2 h (Fig. 5C). In order to demonstrate whether U12 can arrest the cell cycle at G1 by affecting the mTOR/S6K1 pathway, we detected the cell cycle distribution after treatment of rapamycin (mTOR inhibitor) or U12 alone and combination of U12 and rapamycin. Rapamycin and U12 treatment alone for 12 h was found to increase of G1 population by 8% and 22%, respectively. However, combination of rapamycin and U12 caused an attenuation of the U12's effect on G1 cell cycle arrest from 22% to 9%. This was similar to the influence of rapamycin administration alone (Fig. 5D).

Other important regulators of CDKs include a family of inhibitory proteins known as CDKIs. This family includes p21, p27, and p16. These CDKIs can bind and negatively regulate the activity of cyclin-CDK complexes. The present results revealed that U12 treatment can cause over-expression of p27 (Fig.5B) without any noticeable change in p21 or p16 (data not shown). The molecular alterations associated with U12 were consistent with predictions and found to contribute to G1 cell cycle arrest.

### Animal testing

**Figure 6:**
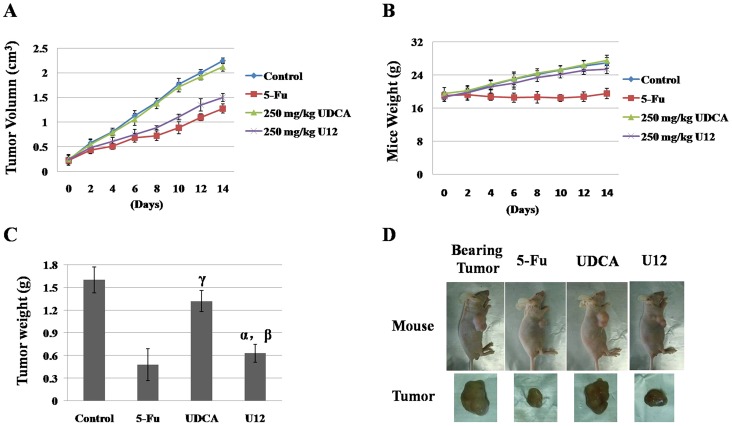
Anti-tumor effects of U12 in tumor xenograft mouse models. Male nude mice bearing HepG2 tumors were treated with vehicle control (2%DMSO in maize oil), 5-Fu (30 mg/kg), UDCA (250 mg/kg), or U12 (250 mg/kg) every day for 2 weeks. Each experimental group contained eight mice. (**A**) Tumor growth inhibition by U12. Tumor volumes were measured with vernier calipers and calculated using the following formula: 0.5A×B^2^, where “A” is the long diameter and “B” is the short diameter (cm)). (**B**) At the end of the treatment (14 days), mice were weighed (error bars represent the standard deviation of the mean). (**C**) At the end of the treatment time (14 days), mice were euthanized and tumors were isolated. The masses of these tumors were measured and averaged. (α, *P* = 0.261, U12 relative to 5-Fu treatment; β, *P* = 0.0008, U12 relative to UDCA treatment; γ, P = 0.073, UDCA relative to control.) (**D**) Representative images of mice and tumors after untreated and treated with 30 mg/kg 5-Fu, 250 mg/kg UDCA and 250 mg/kg U12.

Tumor xenograft model studies were conducted to examine the effects of U12 in *vivo*. HepG2 cells were subcutaneously implanted into 6-week-old male nude mice. After the tumors became palpable, which took just under 2 weeks, the mice were treated intraperitoneally with vehicle control (2% dimethyl sulfoxide (DMSO) in maize oil), 250 mg/kg UDCA, 250 mg/Kg U12 or 30 mg/kg 5-Fu every day for the next two weeks (Fig. 6A&B). Tumor volume was measured three times each week over the course of the 2-week drug treatment, and mice treated with U12 showed significantly less tumor growth than those treated with vehicle when observed after 1-week of treatment (Fig.6A). The weights of the excised tumors confirmed that 250 mg/kg U12 could inhibit tumor growth to an extent similar to that of 5-Fu but greater than that of 250 mg/kg UDCA (Fig. 6C, (α, *P* = 0.261, U12 compared with 5-Fu treatment; β, *P* = 0.0008, U12 relative to UDCA treatment; γ, *P* = 0.073, UDCA relative to control.)). Representative photographs of mice and tumors are shown in Figure 6D and S2 Figure.

Mice treated with 250 mg/kg U12 did not show significantly lower body weight than that of mice treated with 30 mg/kg 5-Fu (Fig. 6B). The weights of mice treated with U12 remained steady over the 2-week treatment period (Fig. 6B).

## Discussion and Conclusions

HCC is a major type of lethal malignant tumor. Chronic hepatitis B and C, alcoholism, and obesity may predispose individuals to cirrhosis, which is a primary risk factor for the development of HCC [25]. No matter what kind of therapeutic strategy is in use, patients with HCC still have poor prognosis and experience many side effects. Developing an efficient chemotherapeutic agent for HCC that involves no toxicity or drug resistance is a top-priority task.

In the present study, 20 different UDCA derivatives were synthesized by esterification at the position of –COOH and through esterification and oxidation at positions 3 and 7-OH (Fig. 1 and S1 File). Of these, U12, derivative modified from UDCA through methyl-esterification at position –COOH and through acetylization at 7-OH, exhibited considerable anticancer activity with no obvious side effects. Under the same conditions and at the same concentration, UDCA only moderately inhibited cell proliferation, showing less than 55% and 80% the effect of U12 in SMMC-7721 and HepG2, respectively (Fig. 2A&B). The results of the present work are consistent with those of previous investigations, which showed that bile acids with different chemical structures and concentrations exhibit different levels of biological activity [26].

The structures of the other 19 derivatives were compared to those of U12. Like U12, U1 was found to lack an acetyl group at 7-OH. U1 has almost no cytotoxicity toward the two liver cancer cell lines and normal liver cells. These results indicate that the presence of acetyl at 7-OH may be associated with the promotion of cell death. U11 and U13 differ from U12 in the presence or absence of the acetyl groups at 3-OH and 7-OH (Fig. 1). These two compounds were found to be inactive against HCC cell proliferation, especially in the HepG2 cell line, and to be more toxic to normal liver cells than U12, indicating that the acetyl group at 3-OH is not necessary to anticancer action even in the presence of the acetyl group at 7-OH. Modification at 7-OH and 3-OH may also influence the activity of U12. This suggests that further investigation of the optimization of this compound's chemical structure is merited.

Results of MetaDrug analysis (Table 1 and S1 Figure) suggest that these proteins are involved in the pathways that regulate the cell cycle, especially transition out of stage G1. The flow cytometric cell cycle analysis performed in the present study confirmed the effect of U12 on G1 phase arrest (Fig. 5A&C). In order to clarify the exact related-pathways involved in the U12-induced G1 phase cell cycle arrest, comparative proteomic approach was then applied. The 2D results and predictions from MetaDrug, together indicated that there are four altered proteins related to cell proliferation, including up-regulation of lamin A/C, EF 2b (partial) and down-regulation of S6K1 and FBP1. Alterations in the concentrations of these four proteins were consistent with the predictions made using MetaDrug and with the effects of U12 on G1 phase arrest (Fig. 4C).

The retinoblastoma protein (Rb) is an important tumor suppressor. It is key to regulation of the cell cycle in a phosphorylation-dependent manner. Hypophosphorylated Rb (p-Rb) was found to be anchored in the nucleus by the interaction with lamin A/C complexes [27]. Proteomic examination showed that the up-regulation of lamin A/C (Fig. 4A–C) may be a compensatory response to the U12-induced decreases in p-Rb (Fig. 4C&5C). EF-2b, one type of EF-2, was reported to be essential to protein synthesis and further cell growth. The increased partial components of EF-2B indicated that levels of functional EF-2B were low, which may have interrupted cell progression. FBP1 is over-expressed in human HCC. The absence of this protein has been reported to reduce the rate of cell proliferation and increase sensitivity to apoptosis [28]. S6K1 is a downstream target of the mTOR, and the mTOR/S6K1 pathway plays an essential role in normal cellular functions, including protein translation, synthesis, stability, cell proliferation, cell cycle progression, and cell survival [29]. Three phosphorylation sites have been identified in S6K1 [30]. The site at Thr389 is essential to the function of S6K1, and activated mTOR can phosphorylate S6K1 at the Thr389 residue, causing phosphorylation and recruitment of the 40S ribosomal unit and finally enhancing the translation of mRNAs, including elongation factors and ribosomal proteins [31].

mTOR is a 289 kDa serine/threonine kinase. The mTOR complex 1 (mTORC1) consists of mTOR, raptor, and mLST8. This complex can regulate cell growth through two important downstream targets, eukaryotic translation initiation factor 4E binding protein1 (4EBP1) and ribosomal S6 kinase1 (S6K1) [29]. The mTOR complex 2 (mTORC2) contains mTOR, rictor, and mLST8. This shows that it can increase the phosphorylation of Akt [32]. Several signaling cascades associated with serine/threonine kinases can regulate the function of mTOR. These include PI3K/AKT kinase pathway and mitogen-activated protein kinase (MAPK) pathway [33]. Many observations show that deregulations of mTOR signaling are usually related to tumorigenesis, angiogenesis, tumor growth and metastasis [34], [35]. The mTOR inhibitors exhibited long-acting tumor suppression in clinical trials. Temsirolimus has been approved by the U.S. Food and Drug Administration (FDA) for treatment of renal-cell carcinoma and mantle-cell lymphoma [24]. RAD001 has shown promise against HCC and phase III studies are expected soon [36]. mTOR inhibitors are not only suitable for use as single therapy in patients but also they can enhance the activity of other anticancer drugs [37]. This is the case with temsirolimus in combination with clofarabine in older patients with advanced AML [24] and temsirolimus in combination with cixutumumab in refractory tumors in the Ewing's sarcoma family [23].

Rapamycin, an mTOR-targeting-molecule, is an approved mTOR inhibitor drug. It has been reported that rapamycin can bind to the intracellular protein FKBP12 to generate a drug-receptor complex and inhibit the kinase activity of mTORC1. The mTORC1/S6K1 signaling pathway has been found to have been activated in several cancer cell lines, and mTORC1 inhibitors have been shown to be effective anticancer agents. For this reason, the current work focuses on the signaling pathways related to mTORC1/S6K1 and G1 cell cycle arrest. Dephosphorylation of mTOR at Ser2448 and S6K1 at Ser371 and Thr389 was observed upon exposure to U12 (Fig. 5C&D). To determine whether U12 can arrest the cell cycle at G1 by affecting the mTORC1/S6K1 pathway, the cell cycle distribution was assessed by administration of rapamycin and combination of rapamycin and U12. Both U12 and rapamycin were found to induce G1 cell cycle arrest, but 20 nM rapamycin appeared to antagonize 50 µM U12 action, showing rescue the U12-involved G1 cell cycle arrest (Fig. 5B). In this way, according to the previous reports about rapamycin and the current results (Fig. 5A–D), U12 was inferred to work through the mTORC1/S6K1 pathway, which was similar to rapamycin. However, it still requires further experiments. In addition, previous studies have demonstrated that rapamycin can decrease the translation rate and stability of cyclinD1 in an mTOR-dependent manner [38]. This induces mTOR-related inhibition of G1 cell cycle progression. The fact that cyclin D plays its role during the early stages of G1 is also consistent with the suppression of Rb activity and the abrogation of the Cdk inhibitor p27 [39]. The phosphorylation state of Rb is related to its repressive activity and it is controlled by the cyclins in families D and E and their corresponding CDKs. To investigate the U12-induced intracellular signaling, the level of phosphorylation of Rb, the levels of expression of cyclin D1, CDK4/6, and p27 were analyzed using Western blotting. U12 has been found to dephosphorylate p-Rb at Ser795 and Ser807, decrease cyclin D1 and CDK4/6 levels, and induce over-expression of p27 in SMMC-7721 cells, which were also consistent with rapamycin's action on G1 arrest. Currently, it is not obvious whether the effects of U12 on S6K1 phosphorylation, cyclin D1 down-regulation, or p27 over-expression in HCC cells are mediated by a linear, split, or parallel pathway. It is here estimated that U12 can arrest the cell cycle at G1 by affecting the mTOR/S6K1 pathway, cyclinD1/CDK2/4 complex, and inducing p27 expression. However, this analysis still suggested that U12's molecular mechanism on G1 arrest was similar to rapamycin and this merits further investigation.

The anti-proliferative activity of U12 was found to be associated with the induction of apoptosis in SMMC-7721 cells, as indicated by in *vitro* evidence of increased caspase-8 and caspase-3 activity and PARP cleavage (Fig. 3E–I). U12 induced-apoptosis was here found to be rescued by 50 µM broad Z-VAD-fmk (spectrum caspase inhibitor) and 20 µM Z-IETD-fmk (specific inhibitors of caspase-8) (Fig. 3C&D), demonstrating the activation of both intrinsic and extrinsic apoptotic pathways. However, the earlier response of caspase-8 at lower concentrations of U12 (Fig. 3G) suggests that U12 treatment can evoke SMMC-7721 cell death primarily beginning with an extrinsic apoptotic pathway.

250 mg/kg U12-treated mice showed considerable antitumor effects but no significant toxic effects, as indicated by decrease in tumor size and weight, maintenance of mice body weight and lack of obvious organ damage (data not shown) during the treatment period (Fig. 6A–D). And the same concentration of UDCA were not examined the obvious toxic effects toward mice tumors. Moreover, 250 mg/kg U12 exhibited a similar effect on inhibition of tumor growth, but it showed a better ability to help mice maintain their weight than 30 mg/kg 5-Fu (Fig. 6B&C).

In summary, 20 UDCA analogues were synthesized through modification at 3-OH, 7-OH, and –COOH. The lead compound, U12, was identified. It displayed considerable and effective anticancer activity in liver cancer cell lines *in vitro* and in mice *in vivo*, and it did not show any obvious adverse effects. A preliminary structure-activity relationship analysis of U12 suggested that acetylization at 7-OH of UDCA was critical to its anticancer activity and to its low toxicity to normal liver cells.

This compound has a simple and concise structural motif and is easily synthesized and optimized. The results of the current study indicate that multiple pathways participate in U12-induced anti-proliferative and pro-apoptotic action, including the mTOR/S6K1, cyclinD1/CDK2/4 signaling pathways and caspase-dependent apoptotic pathways. U12, similar to rapamycin, might work through the mTOR/S6K1 pathway. These observations collectively indicated that U12 differs from UDCA and other derivatives and may be a suitable lead for the development of compounds useful in the treatment of HCC.

## Supporting Information

S1 Figure Prediction of the mechanism of U12 anti-cancer actions using MetaDrug.
(TIF)

S2 Figure Images of untreated tumors and tumors treated daily with indicated drugs for 2 weeks. Male nude mice bearing HepG2 tumors were treated with vehicle control (2%DMSO in maize oil), 30 mg/Kg 5-Fu, 250 mg/Kg UDCA or 250 mg/Kg U12. Each experimental group contained eight mice.
(TIF)

S1 File Experimental synthesis of the 20 different UDCA derivatives.
(DOC)

## References

[1] Herszenyi L, Tulassay Z (2010) Epidemiology of gastrointestinal and liver tumors. Eur Rev Med Pharmacol Sci 14:249–258. 20496531

[2] Aravalli RN, Cressman EN, Steer CJ (2013) Cellular and molecular mechanisms of hepatocellular carcinoma: an update. Arch Toxicol 87:227–247. 23007558 10.1007/s00204-012-0931-2

[3] Nojiri S, Nakao H, Sugauchi F, Miyaki T, Senda K, et al (2009) Effect of ursodeoxycholic acid on serum liver enzymes and bile acid metabolism in chronic active hepatitis C virus infection. Hepatol Res 39:21–30. 18721155 10.1111/j.1872-034X.2008.00406.x

[4] Pardi DS, Loftus EV Jr, Kremers WK, Keach J, Lindor KD (2003) Ursodeoxycholic acid as a chemopreventive agent in patients with ulcerative colitis and primary sclerosing cholangitis. Gastroenterology 124:889–893. 12671884 10.1053/gast.2003.50156

[5] Kappler M, Espach C, Schweiger-Kabesch A, Lang T, Hartl D, et al. (2012) Ursodeoxycholic acid therapy in cystic fibrosis liver disease - a retrospective long-term follow-up case-control study. Aliment Pharmacol Ther. 10.1111/j.1365-2036.2012.05177.x 22670841

[6] Sola S, Ma X, Castro RE, Kren BT, Steer CJ, et al (2003) Ursodeoxycholic acid modulates E2F-1 and p53 expression through a caspase-independent mechanism in transforming growth factor beta1-induced apoptosis of rat hepatocytes. J Biol Chem 278:48831–48838. 14514686 10.1074/jbc.M300468200

[7] Amaral JD, Castro RE, Steer CJ, Rodrigues CM (2009) p53 and the regulation of hepatocyte apoptosis: implications for disease pathogenesis. Trends Mol Med 15:531–541. 19822456 10.1016/j.molmed.2009.09.005

[8] Amaral JD, Castro RE, Sola S, Steer CJ, Rodrigues CM (2007) p53 is a key molecular target of ursodeoxycholic acid in regulating apoptosis. J Biol Chem 282:34250–34259. 17881359 10.1074/jbc.M704075200

[9] Alberts DS, Martinez ME, Hess LM, Einspahr JG, Green SB, et al (2005) Phase III trial of ursodeoxycholic acid to prevent colorectal adenoma recurrence. J Natl Cancer Inst 97:846–853. 15928305 10.1093/jnci/dji144

[10] Khare S, Mustafi R, Cerda S, Yuan W, Jagadeeswaran S, et al (2008) Ursodeoxycholic acid suppresses Cox-2 expression in colon cancer: roles of Ras, p38, and CCAAT/enhancer-binding protein. Nutr Cancer 60:389–400. 18444174 10.1080/01635580701883003

[11] Asciutti S, Castellani D, Nardi E, Morelli O, Clementi M, et al (2009) A new amino acid derivative of ursodeoxycholate, (N-L-Glutamyl)-UDCA (UDCA-Glu), to selectively release UDCA in the colon. Anticancer Res 29:4971–4979. 20044604

[12] Fiorucci S, Antonelli E, Distrutti E, Mencarelli A, Farneti S, et al (2004) Liver delivery of NO by NCX-1000 protects against acute liver failure and mitochondrial dysfunction induced by APAP in mice. Br J Pharmacol 143:33–42. 15345658 10.1038/sj.bjp.0705780 PMC1575257

[13] Fiorucci S, Antonelli E, Morelli O, Mencarelli A, Casini A, et al (2001) NCX-1000, a NO-releasing derivative of ursodeoxycholic acid, selectively delivers NO to the liver and protects against development of portal hypertension. Proc Natl Acad Sci U S A 98:8897–8902. 11447266 10.1073/pnas.151136298 PMC37532

[14] Im EO, Choi YH, Paik KJ, Suh H, Jin Y, et al (2001) Novel bile acid derivatives induce apoptosis via a p53-independent pathway in human breast carcinoma cells. Cancer Lett 163:83–93. 11163111 10.1016/s0304-3835(00)00671-6

[15] Choi YH, Im EO, Suh H, Jin Y, Yoo YH, et al (2003) Apoptosis and modulation of cell cycle control by synthetic derivatives of ursodeoxycholic acid and chenodeoxycholic acid in human prostate cancer cells. Cancer Lett 199:157–167. 12969788 10.1016/s0304-3835(03)00351-3

[16] Im E, Choi SH, Suh H, Choi YH, Yoo YH, et al (2005) Synthetic bile acid derivatives induce apoptosis through a c-Jun N-terminal kinase and NF-kappaB-dependent process in human cervical carcinoma cells. Cancer Lett 229:49–57. 16157218 10.1016/j.canlet.2004.11.055

[17] Yang F, Huo X-s, Yuan S-x, Zhang L, Zhou W-p, et al (2013) Repression of the Long Noncoding RNA-LET by Histone Deacetylase 3 Contributes to Hypoxia-Mediated Metastasis. Molecular Cell 49:1083–1096. 23395002 10.1016/j.molcel.2013.01.010

[18] Xu Y, Chiu JF, He QY, Chen F (2009) Tubeimoside-1 exerts cytotoxicity in HeLa cells through mitochondrial dysfunction and endoplasmic reticulum stress pathways. J Proteome Res 8:1585–1593. 19215086 10.1021/pr801001j

[19] Li Y, Jin M, Shao S, Huang W, Yang F, et al (2014) Small-sized polymeric micelles incorporating docetaxel suppress distant metastases in the clinically-relevant 4T1 mouse breast cancer model. BMC Cancer 14:329. 24885518 10.1186/1471-2407-14-329 PMC4023534

[20] Chen T, Xu Y, Guo H, Liu Y, Hu P, et al (2011) Experimental therapy of ovarian cancer with synthetic makaluvamine analog: in vitro and in vivo anticancer activity and molecular mechanisms of action. PLoS One 6:e20729. 21673964 10.1371/journal.pone.0020729 PMC3108973

[21] Im E, Martinez JD (2004) Ursodeoxycholic acid (UDCA) can inhibit deoxycholic acid (DCA)-induced apoptosis via modulation of EGFR/Raf-1/ERK signaling in human colon cancer cells. J Nutr 134:483–486. 14747693 10.1093/jn/134.2.483

[22] Paolini M, Pozzetti L, Montagnani M, Potenza G, Sabatini L, et al (2002) Ursodeoxycholic acid (UDCA) prevents DCA effects on male mouse liver via up-regulation of CYP [correction of CXP] and preservation of BSEP activities. Hepatology 36:305–314. 12143038 10.1053/jhep.2002.34939

[23] Naing A, LoRusso P, Fu S, Hong DS, Anderson P, et al (2012) Insulin growth factor-receptor (IGF-1R) antibody cixutumumab combined with the mTOR inhibitor temsirolimus in patients with refractory Ewing's sarcoma family tumors. Clin Cancer Res 18:2625–2631. 22465830 10.1158/1078-0432.CCR-12-0061 PMC3875297

[24] Amadori S, Stasi R, Martelli AM, Venditti A, Meloni G, et al (2012) Temsirolimus, an mTOR inhibitor, in combination with lower-dose clofarabine as salvage therapy for older patients with acute myeloid leukaemia: results of a phase II GIMEMA study (AML-1107). Br J Haematol 156:205–212. 22082314 10.1111/j.1365-2141.2011.08940.x

[25] Aravalli RN, Cressman EN, Steer CJ (2012) Cellular and molecular mechanisms of hepatocellular carcinoma: an update. Arch Toxicol. 10.1007/s00204-012-0931-2 23007558

[26] Milovic V, Teller IC, Faust D, Caspary WF, Stein J (2002) Effects of deoxycholate on human colon cancer cells: apoptosis or proliferation. Eur J Clin Invest 32:29–34. 11851724 10.1046/j.0014-2972.2001.00938.x

[27] Markiewicz E, Dechat T, Foisner R, Quinlan RA, Hutchison CJ (2002) Lamin A/C binding protein LAP2alpha is required for nuclear anchorage of retinoblastoma protein. Mol Biol Cell 13:4401–4413. 12475961 10.1091/mbc.E02-07-0450 PMC138642

[28] Rabenhorst U, Beinoraviciute-Kellner R, Brezniceanu ML, Joos S, Devens F, et al (2009) Overexpression of the far upstream element binding protein 1 in hepatocellular carcinoma is required for tumor growth. Hepatology 50:1121–1129. 19637194 10.1002/hep.23098 PMC3474328

[29] Jiang BH, Liu LZ (2008) Role of mTOR in anticancer drug resistance: perspectives for improved drug treatment. Drug Resist Updat 11:63–76. 18440854 10.1016/j.drup.2008.03.001 PMC2519122

[30] Dennis PB, Pullen N, Kozma SC, Thomas G (1996) The principal rapamycin-sensitive p70(s6k) phosphorylation sites, T-229 and T-389, are differentially regulated by rapamycin-insensitive kinase kinases. Mol Cell Biol 16:6242–6251. 8887654 10.1128/mcb.16.11.6242 PMC231627

[31] Faivre S, Kroemer G, Raymond E (2006) Current development of mTOR inhibitors as anticancer agents. Nat Rev Drug Discov 5:671–688. 16883305 10.1038/nrd2062

[32] Barilli A, Visigalli R, Sala R, Gazzola GC, Parolari A, et al (2008) In human endothelial cells rapamycin causes mTORC2 inhibition and impairs cell viability and function. Cardiovasc Res 78:563–571. 18250144 10.1093/cvr/cvn024

[33] Endo M, Yamamoto H, Setsu N, Kohashi K, Takahashi Y, et al. (2012) Prognostic significance of AKT/mTOR and MAPK pathways and antitumor effect of mTOR inhibitor in NF1-related and sporadic malignant peripheral nerve sheath tumors. Clin Cancer Res. 10.1158/1078-0432.CCR-12-1067 23209032

[34] Huang S, Bjornsti MA, Houghton PJ (2003) Rapamycins: mechanism of action and cellular resistance. Cancer Biol Ther 2:222–232. 12878853 10.4161/cbt.2.3.360

[35] Saito K, Matsumoto S, Yasui H, Devasahayam N, Subramanian S, et al (2012) Longitudinal Imaging Studies of Tumor Microenvironment in Mice Treated with the mTOR Inhibitor Rapamycin. PLoS One 7:e49456. 23185335 10.1371/journal.pone.0049456 PMC3502528

[36] Kudo M (2011) mTOR inhibitor for the treatment of hepatocellular carcinoma. Dig Dis 29:310–315. 21829022 10.1159/000327565

[37] Yang S, Xiao X, Meng X, Leslie KK (2011) A mechanism for synergy with combined mTOR and PI3 kinase inhibitors. PLoS One 6:e26343. 22039466 10.1371/journal.pone.0026343 PMC3198385

[38] Grewe M, Gansauge F, Schmid RM, Adler G, Seufferlein T (1999) Regulation of cell growth and cyclin D1 expression by the constitutively active FRAP-p70s6K pathway in human pancreatic cancer cells. Cancer Res 59:3581–3587. 10446965

[39] Zhang HS, Postigo AA, Dean DC (1999) Active transcriptional repression by the Rb-E2F complex mediates G1 arrest triggered by p16INK4a, TGFbeta, and contact inhibition. Cell 97:53–61. 10199402 10.1016/s0092-8674(00)80714-x

