# Supplementary figures and images for: U12, a UDCA Derivative, Acts as an Anti-Hepatoma Drug Lead and Inhibits the mTOR/S6K1 and Cyclin/CDK Complex Pathways
Source: PLoS One. 2014 Dec 8;9(12):e113479. doi: 10.1371/journal.pone.0113479 (PMC4259312; doi:10.1371/journal.pone.0113479)

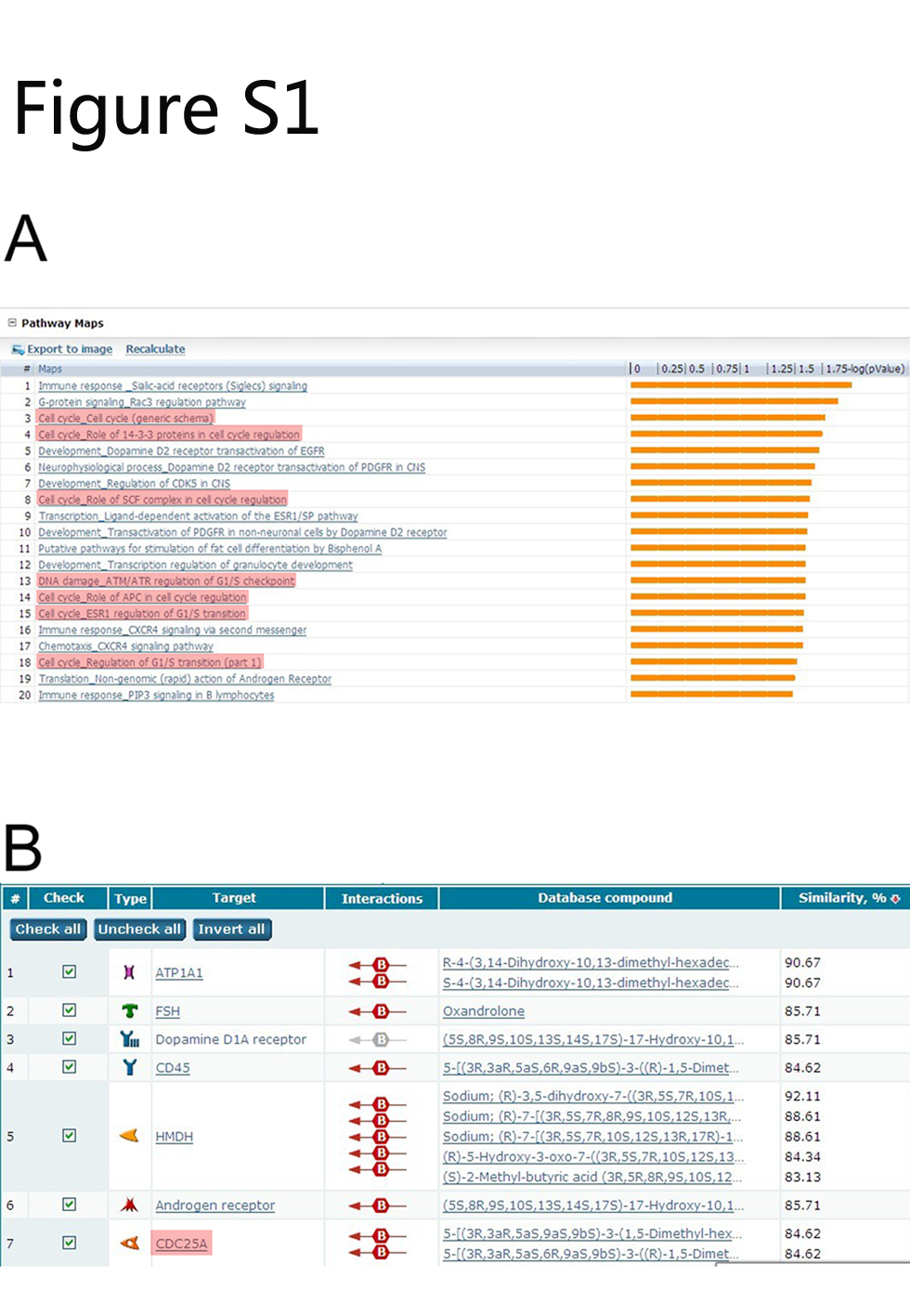

Supplement: S1 Figure — Prediction of the mechanism of U12 anti-cancer actions using MetaDrug. (TIF) [file pone.0113479.s001.tif]

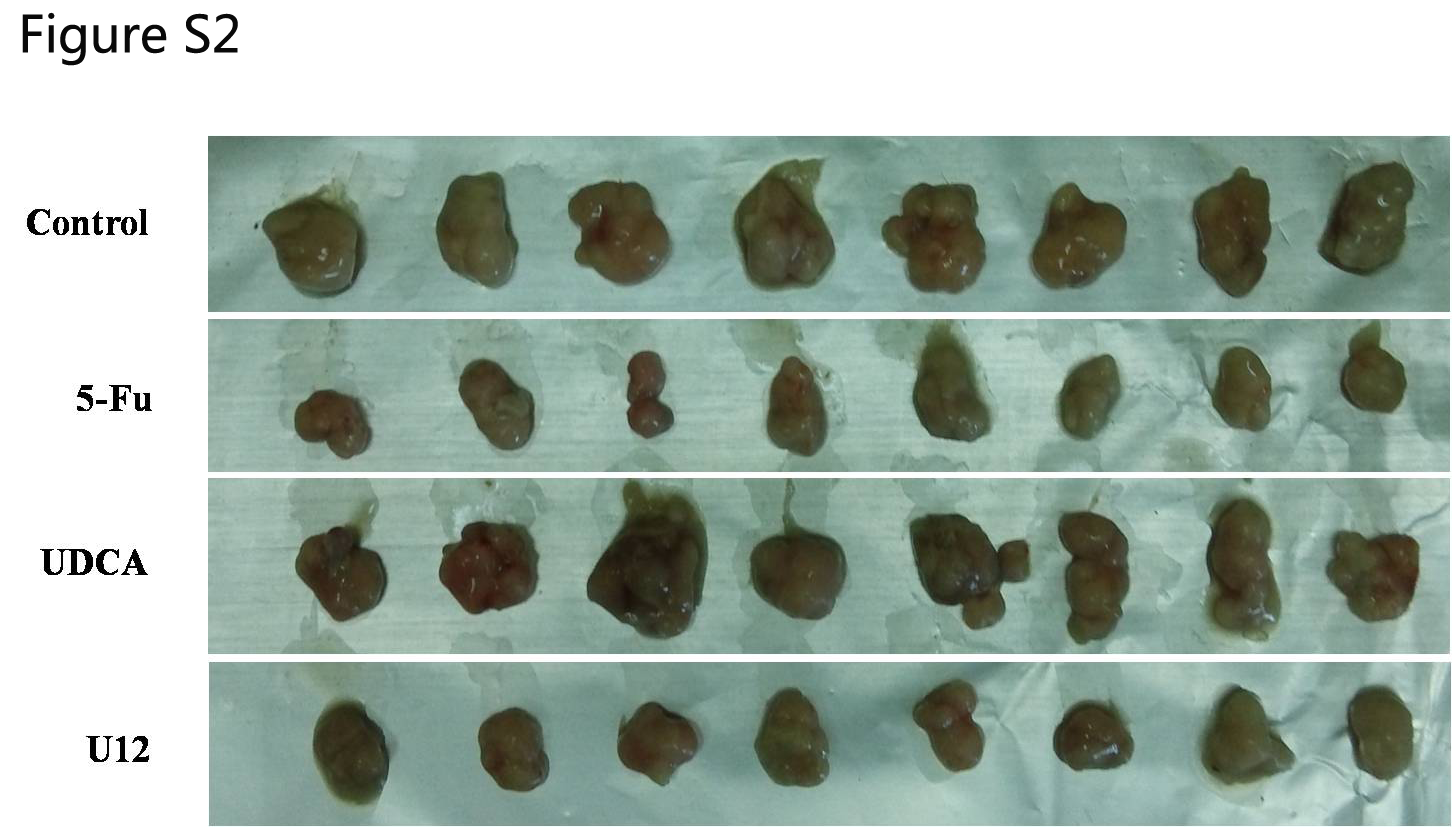

Supplement: S2 Figure — Images of untreated tumors and tumors treated daily with indicated drugs for 2 weeks. Male nude mice bearing HepG2 tumors were treated with vehicle control (2%DMSO in maize oil), 30 mg/Kg 5-Fu, 250 mg/Kg UDCA or 250 mg/Kg U12. Each experimental group contained eight mice. (TIF) [file pone.0113479.s002.tif]
