# Supplementary material for: U12, a UDCA Derivative, Acts as an Anti-Hepatoma Drug Lead and Inhibits the mTOR/S6K1 and Cyclin/CDK Complex Pathways
Source: PLoS One. 2014 Dec 8;9(12):e113479. doi: 10.1371/journal.pone.0113479 (PMC4259312; doi:10.1371/journal.pone.0113479)
Supplement: S1 File — Experimental synthesis of the 20 different UDCA derivatives. (DOC) [file pone.0113479.s003.doc]

**Supplement 1**

**Experimental procedures of Chemistry**

**General method**

NMR spectra were recorded on a Bruker DRX 500 spectrometer. Residual signals of solvent served as internal references (CDCl_3_: δ_H_ 7.26, δ_C_ 77.6; CD_3_OD: δ_H_ 3.31, δ_C_ 49.9). Dichloromethane was distilled from phosphorus pentoxide. Methanol was distilled from magnesium turnings and iodine. Silica gel (300–400 mesh) from Qingdao Haiyang Chemical Cl., Ltd. (China) was used for column chromatography, eluted (unless otherwise stated) with a petroleum ether (PE) (60–90°C)/ethyl acetate mixture or petroleum ether (PE) (60–90°C)/acetone.

**General procedures for preparing U1–3 via esterification**

1.2 equiv volumes of thionyl chloride (217 μL) were added to a stirred solution of UDCA (1.0 g, 2.55 mmol) in corresponding alkanol (methanol, ethanol or n-butanol) at 0°C. After 30 min, the mixtures were stirred at room temperature for 30 minutes and at reflux for an additional 2 h. The resulting mixtures were concentrated in vacuo and the residues were recrystallized with diethyl ether to produce the corresponding products U1–3.

1.04 g (yield 100%) of U1 product was obtained and purified to a white solid. ^1^H NMR (500 MHz, CDCl_3_) δ: 0.77 (s, 3H), 0.99 (d, *J* = 6.5 Hz, 3H), 1.00 (s, 3H), 3.24 (br, 2H), 3.34 (s, 3H). ^13^C NMR (125 MHz, CDCl_3_) δ: 175.1, 71.4, 71.3, 56.3, 55.4, 51.9, 44.1, 43.9, 42.9, 40.6, 39.8, 37.7, 37.6, 35.7, 35.4, 34.4, 31.5, 31.4, 30.6, 29.0, 27.3, 23.8, 21.6, 18.7, 12.5.

1.04 g (yield 98%) of U2 product was obtained, purified as a light yellow paste. ^1^H NMR (500 MHz, CD_3_OD) δ: 0.74 (s, 3H), 0.99 (d, *J* = 6.5 Hz, 3H), 1.00 (s, 3H), 4.13 (m, 2H). ^13^C NMR (125 MHz, CD_3_OD) δ: 176.3, 72.5, 72.3, 61.9, 57.9, 56.9, 45.2, 44.9, 44.5, 42.0, 41.2, 39.1, 38.5, 37.1, 36.6, 35.6, 32.8, 32.6, 31.5, 30.2, 28.4, 24.5, 22.9, 19.5, 15.1, 13.2.

1.07 g (yield 95%) of U3 product was obtained and purified to a light yellow paste. ^1^H NMR (500 MHz, CD_3_OD) δ: 0.74 (s, 3H), 0.99 (d, *J* = 6.5 Hz, 3H), 1.00 (s, 3H), 4.09 (t, *J* = 6.5 Hz, 2H). ^13^C NMR (125 MHz, CD_3_OD) δ: 176.3, 72.6, 72.3, 65.7, 58.0, 57.0, 45.3, 44.9, 44.5, 42.1, 41.2, 39.1, 38.5, 37.1, 36.7, 35.7, 32.8, 32.7, 32.4, 31.6, 30.2, 28.4, 24.6, 22.9, 20.8, 19.6, 14.7, 13.3.

**General procedures for preparing U4 via etherification**

First, 1.2 equivalent volumes of iodomethane (15 μL) were added to a stirred solution of U1 (UDCA methyl ester, 100 mg, 0.246 mmol) and K_2_CO_3_ (2 equiv, 34 mg) in DMF (5 mL) at 0°C. Then the mixture was stirred at room temperature for 12 h, diluted with H_2_O (10 mL), and extracted with 10 mL acetic ester. The resulting extract was purified by silica gel column chromatography (petroleum ether (PE) (60–90°C)/acetic ester = 4:1) to produce light yellow liquid U4 (77.5 mg, yield 75%). ^1^H NMR (500 MHz, CDCl_3_) δ: 0.77 (s, 3H), 0.99 (d, *J* = 6.5 Hz, 3H), 1.00 (s, 3H), 3.22 (s,3H), 3.34 (s, 3H). ^13^C NMR (125 MHz, CDCl_3_) δ: 175.1, 80.5, 71.7, 56.1, 55.9, 55.3, 51.8, 44.1, 44.0, 42.8, 40.5, 39.5, 37.3, 35.6, 35.2, 34.8, 34.1, 31.5, 31.4, 28.9, 27.2, 27.0, 23.8, 21.6, 18.7, 12.5.

**General procedures for preparing U5–10 via oxidation**

Two equiv of PCC were added to a stirred solution of UDCA or the corresponding ester (200 mg) in dichloromethane (20 mL) at room temperature. The mixture was stirred at room temperature for 1 h. The appearance of products was confirmed by TLC. The resulting solution was filtered. The filtrate was concentrated in vacuo and then purified by silica gel column chromatography (petroleum ether (PE) (60–90°C)/acetone = 4:1) to produce U5–10.

78.8 mg (yield 40%) of U5 product was obtained and purified to a light yellow solid. ^1^H NMR (500 MHz, CD_3_OD) δ: 0.68 (s, 3H), 0.99 (d, *J* = 6.5 Hz, 3H), 1.05 (s, 3H), 4.00 (br, 1H). ^13^C NMR (125 MHz, CD_3_OD) δ: 215.5, 176.4, 71.9, 61.8, 56.7, 51.1, 50.8, 47.9, 46.8, 44.8, 44.2, 40.8, 38.6, 36.9, 36.7, 35.6, 32.7, 32.6, 31.1, 29.7, 26.2, 23.9, 23.2, 19.3, 12.9.

88.2 mg (yield 45%) of U6 product was obtained and purified to a light yellow solid. ^1^H NMR (500 MHz, CD_3_OD) δ: 0.68 (s, 3H), 1.02 (d, *J* = 6.0 Hz, 3H), 1.36 (s, 3H). ^13^C NMR (125 MHz, CD_3_OD) δ: 213.2, 209.8, 175.4, 55.2, 51.0, 49.7, 49.2, 44.9, 43.1, 42.8, 39.1, 36.5, 35.5, 35.4, 31.2, 30.8, 28.2, 24.7, 22.4, 22.2, 21.6, 17.8, 11.4.

168.3 mg (yield 85%) of U7 product was obtained and purified to a light yellow solid. ^1^H NMR (500 MHz, CDCl_3_) δ: 0.69 (s, 3H), 0.93 (d, *J* = 6.0 Hz, 3H), 1.31 (s, 3H), 3.67 (s, 3H). ^13^C NMR (125 MHz, CDCl_3_) δ: 211.5, 210.5, 174.9, 55.1, 51.8, 49.9, 49.2, 48.1, 45.4, 43.3, 43.2, 43.0, 39.2, 37.1, 35.8, 35.5, 31.3, 31.3, 30.7, 28.6, 25.1, 22.8, 22.5, 18.7, 12.4.

158.5 mg (yield 80%) of U8 product was obtained and purified to a white solid. ^1^H NMR (500 MHz, CDCl_3_) δ: 0.58 (s, 3H), 0.82 (d, *J* = 6.0 Hz, 3H), 1.20 (s, 3H), 4.01 (m, 2H). ^13^C NMR (125 MHz, CDCl_3_) δ: 211.7, 210.8, 174.6, 60.6, 58.5, 55.1, 49.9, 49.2, 48.1, 45.4, 43.3, 43.2, 43.0, 39.2, 37.1, 35.8, 35.5, 31.6, 31.3, 28.6, 25.1, 22.8, 22.5, 18.7, 14.6, 12.4.

63.7 mg (yield 32%) of U9 product was obtained and purified to a light yellow paste. ^1^H NMR (500 MHz, CD_3_OD) δ: 0.65 (s, 3H), 0.86 (d, *J* = 5.0 Hz, 3H), 1.19 (s, 3H), 3.97 (m, 3H). ^13^C NMR (125 MHz, CD_3_OD) δ: 214.1, 175.1, 70.3, 64.3, 56.1, 55.5, 45.1, 43.8, 43.2, 42.9, 40.3, 39.5, 36.9, 36.8, 36.4, 35.6, 34.5, 31.4, 31.2, 30.9, 29.7, 28.6, 26.9, 22.0, 21.9, 19.2, 17.9, 13.0.

79.3 mg (yield 40%) of U10 product was obtained and purified to a white solid. ^1^H NMR (500 MHz, CDCl_3_) δ: 0.62 (s, 3H), 0.86 (d, *J* = 6.0 Hz, 3H), 1.24 (s, 3H), 3.99 (t, *J* = 6.5 Hz, 2H). ^13^C NMR (125 MHz, CDCl_3_) δ: 211.6, 210.7, 174.7, 64.6, 55.2, 49.9, 49.2, 48.2, 45.4, 43.3, 43.2, 43.0, 39.2, 37.1, 35.8, 35.6, 31.7, 31.4, 31.1, 28.6, 25.2, 22.8, 22.5, 19.6, 18.8, 14.1, 12.5.

**General procedures for preparing U11–13 via acyl esterification**

A drop of H_2_SO_4_ was added to a stirred solution of U1 (UDCA methyl ester, 200 mg, 0.493 mmol) in acetic anhydride (4 equiv). When the mixture was stirred at room temperature for 30 min, the appearances of three products were checked by TLC. After 1 h, the solution was diluted with H_2_O (20 mL) and extracted with 20 mL acetic ester. The resulting extract was dried, concentrated in vacuo and purified by silica gel column chromatography (petroleum ether (PE) (60–90°C)/acetic ester = 5:1) to give U11-13.

65.6 mg (yield 30%) of U11 product was obtained and purified to a white solid. ^1^H NMR (500 MHz, CDCl_3_) δ: 1.92 (s, 3H), 3.56 (s, 3H), 4.56 (br, 1H), 5.23 (s, 1H). ^13^C NMR (125 MHz, CDCl_3_) δ: 174.9, 170.7, 74.2, 71.3, 56.2, 55.3, 51.7, 44.0, 43.9, 42.7, 40.5, 39.5, 37.3, 35.6, 35.0, 34.4, 33.5, 31.3, 28.9, 27.2, 26.8, 23.7, 21.7, 21.6, 18.7, 12.5.

54.6 mg (yield 25%) of U12 product was obtained, and purified to a light yellow paste. ^1^H NMR (500 MHz, CDCl_3_) δ: 1.98 (s, 3H), 3.65 (s, 3H), 4.77 (br, 1H). ^13^C NMR (125 MHz, CDCl_3_) δ: 175.0, 171.0, 74.3, 71.4, 55.6, 55.3, 51.8, 43.9, 42.6, 40.3 39.7, 37.4, 35.6, 35.2, 34.3, 33.4, 31.4, 30.4, 30.1, 28.8, 26.0, 23.6, 22.2, 21.5, 18.7, 12.4.

52.6 mg (yield 22%) of U13 product was obtained and purified to a white solid. ^1^H NMR (500 MHz, CDCl_3_) δ: 1.90 (s, 3H), 1.94 (s, 3H), 3.58 (s, 3H), 4.68 (br, 2H). ^13^C NMR (125 MHz, CDCl_3_) δ:174.8, 170.7, 73.8, 55.5, 55.3, 51.7, 43.9, 42.4, 40.2, 39.7, 35.5, 34.8, 34.3, 33.2, 31.3, 31.1, 30.0, 28.7, 26.7, 25.9, 23.5, 22.0, 21.6, 21.5, 18.7, 12.4.

**General procedures for preparing U14 via oxidation**

Two equiv of PCC were added to a stirred solution of U11 (25 mg, 0.056 mmol) in dichloromethane (1 mL) at room temperature. When the mixture was stirred at room temperature for 4 h, the appearance of only one product and the disappearance of the starting material were confirmed by TLC. The resulting solution was filtered, the filtrate was concentrated in vacuo, and the produced was then purified by silica gel column chromatography (petroleum ether (PE) (60–90°C)/acetone = 8:1) to produce U14.

22.9 mg (yield 92%) of U14 product was obtained and purified to a white solid. ^1^H NMR (500 MHz, CDCl_3_) δ: 0.65 (s, 3H), 0.92 (d, *J* = 6.5 Hz, 3H), 1.21 (s, 3H), 2.00 (s, 3H), 3.67 (s, 3H), 4.68 (br, 1H). ^13^C NMR (125 MHz, CDCl_3_) δ: 209.2, 175.1, 171.0, 73.3, 55.2, 51.9, 49.9, 49.3, 46.2, 45.7, 43.1, 43.0, 39.3, 35.6, 34.2, 33.5, 31.4, 30.1, 28.7, 26.4, 25.2, 23.4, 22.1, 21.7, 18.8, 12.5.

**General procedures for preparing U15–16 via acyl esterification**

Two equiv of benzoic acid and a drop of H_2_SO_4_ were added to a stirred solution of U1 (UDCA methyl ester, 200 mg, 0.493 mmol) in 10 mL acetone. After the mixture was stirred at room temperature for 30 min, the appearances of two products were confirmed by TLC. After 1 h, the mixture was quenched. The resulting solution was diluted with 30 mL H_2_O and extracted with acetic ester (30 mL). The extract was dried, concentrated in vacuo, and purified by silica gel column chromatography PE (60–90°C)/acetic ester = 8:1) to produce U15-16.

87.1 mg (yield 35%) of U15 product was obtained and purified as a white solid. ^1^H NMR (500 MHz, CDCl_3_) δ: 0.61 (s, 3H), 0.86 (d, *J* = 6.5Hz, 3H), 0.91 (s, 3H), 3.59 (s, 3H), 7.35 (m, 2H), 7.47 (m, 1H), 7.96 (d, *J* = 7.5 Hz, 1H). ^13^C NMR of U15 (125 MHz, CDCl_3_) δ: 175.2, 166.5, 133.2, 131.2, 129.9, 129.9, 128.7, 128.7, 74.8, 71.6, 56.2, 55.3, 51.9, 44.2, 44.2, 42.8, 40.5, 39.6, 37.2, 35.7, 35.1, 34.6, 33.7, 31.4, 31.4, 29.0, 27.3, 27.0, 23.8, 21.7, 18.8, 12.6.

74.6 mg (yield 30%) of U16 product was obtained and purified to a white solid. ^1^H NMR (500 MHz, CDCl_3_) δ: 0.64 (s, 3H), 0.82 (d, *J* = 6.5 Hz, 3H), 0.96 (s, 3H), 3.53 (s, 3H), 7.34 (m, 4H), 7.45 (m, 2H), 7.95 (d, *J* = 7.5 Hz, 4H). ^13^C NMR of U16 (125 MHz, CDCl_3_) δ: 175.0, 166.4, 166.4, 133.2, 131.3, 131.1, 130.0, 128.7, 74.8, 74.6, 55.6, 55.3, 51.9, 44.0, 42.6, 40.6, 40.3, 39.9, 35.6, 35.0, 34.5, 33.4, 31.3, 28.9, 27.0, 26.6, 23.8, 21.7, 18.8, 12.6.

**General procedures for preparing U17–18 via sulfonation**

Two equiv each of methanesulfonyl chloride and triethylamine were added to a stirred solution of U1 (UDCA methyl ester, 200 mg, 0.493 mmol) in 10 mL CH2Cl2 at 0°C. After the mixture was stirred at 0°C for 30 min, the appearances of two products were confirmed by TLC. After 1 h, the mixture was quenched. The resulting solution was diluted with 30 mL H_2_O and extracted with dichloromethane (20 mL). The extract was dried, concentrated in vacuo, and purified by silica gel column chromatography PE (60–90°C)/acetic ester = 5:1) to produce U17-18.

70.8 mg (yield 30%) of U17 product was obtained and purified to a white solid. ^1^H NMR (500 MHz, CDCl_3_) δ: 0.68 (s, 3H), 0.93 (d, *J* = 6.0Hz, 3H), 0.96 (s, 3H), 3.00 (s, 3H), 3.67 (s, 3H). ^13^C NMR (125 MHz, CDCl_3_) δ: 175.1, 82.5, 71.4, 56.1, 55.3, 51.9, 44.1, 44.0, 42.9, 40.4, 39.6, 39.2, 37.3, 35.6, 35.0, 34.6, 34.3, 31.4, 29.0, 28.1, 27.2, 23.6, 21.6, 18.8, 12.5.

123.4 mg (yield 45%) of U18 product was obtained and purified to a white solid. ^1^H NMR (500 MHz, CDCl_3_) δ: 2.93 (s, 3H), 2.94 (s, 3H), 3.58 (s, 3H). ^13^C NMR (125 MHz, CDCl_3_) δ: 174.9, 82.6, 81.6, 55.2, 55.0, 51.8, 44.1, 42.6, 41.2, 40.4, 39.9, 39.8, 39.1, 35.5, 34.5, 34.0, 33.9, 31.3, 31.2, 28.7, 28.0, 26.3, 23.3, 21.6, 18.7, 12.4.

**General procedures for preparing U19 via oxidation**

Two equiv of PCC were added to a stirred solution of U17 (30 mg, 0.062 mmol) in 1 mL CH_2_Cl_2_. After the mixture had been stirred at room temperature for 4 h, the appearance of only one product and the disappearance of the starting material were confirmed by TLC. After the mixture was quenched, the resulting solution was filtered, the filtrate was concentrated in vacuo and then purified by silica gel column chromatography (petroleum ether (PE) (60–90°C)/acetone = 8:1) to produce U19.

26.9 mg (yield 90%) of U19 product was obtained and purified to a white solid. ^1^H NMR (500 MHz, CDCl_3_) δ: 2.97 (s, 3H), 3.64 (s, 3H). ^13^C NMR (125 MHz, CDCl_3_) δ: 212.0, 175.1, 81.2, 55.1, 51.9, 49.8, 49.2, 46.3, 45.4, 43.1, 43.0, 39.4, 39.2, 35.6, 35.3, 34.4, 34.1, 31.4, 31.3, 28.6, 27.8, 25.1, 23.2, 22.1, 18.7, 12.4.

**General procedures for preparing U20 via acyl esterification**

Two equivalent volumes of acetic anhydride and a drop of H_2_SO_4_ were added to a stirred solution of U17 (30 mg, 0.062 mmol). The mixture was then stirred at room temperature for 4 h. The resulting extract was purified by silica gel column chromatography (petroleum ether (PE) (60–90°C)/acetic ester = 10:1) to give U20.

27.7 mg (yield 85%) of U20 product was obtained. It was then purified to a white solid. ^1^H NMR (500 MHz, CDCl_3_) δ: 1.96 (s, 3H), 2.99 (s, 3H), 3.63 (s, 3H). ^13^C NMR (125 MHz, CDCl_3_) δ:175.0, 171.0, 82.0, 73.7, 55.6, 55.3, 51.9, 43.9, 42.6, 40.2, 39.7, 39.3, 35.6, 34.8, 34.2, 33.0, 31.4, 31.3, 28.8, 28.1, 26.0, 23.4, 22.2, 21.6, 18.7, 12.4.
